# Rotational Spectroscopy as a Tool to Characterize Sweet Taste: The Study of Dulcin

**DOI:** 10.1002/open.202400159

**Published:** 2024-07-25

**Authors:** Gabriela Juarez, Elena R. Alonso, Raúl Aguado, Iker León

**Affiliations:** ^1^ Grupo de Espectroscopia Molecular (GEM) Edificio Quifima Laboratorios de Espectroscopia y Bioespectroscopia Unidad Asociada CSIC Parque Científico Uva Universidad de Valladolid Paseo de Belén 5 47011 Valladolid Spain

**Keywords:** Artificial Sweetener, Theory of Sweetness, Dulcin, Microwave Spectroscopy, Structure

## Abstract

According to old theories of sweetness, the perception of sweet substances is closely linked to the arrangement of atoms within them. To assess the validity of these theories, we conducted an analysis of the structure of the artificial sweetener dulcin for the first time, utilizing microwave spectroscopy and a laser ablation source. These techniques have enabled the identification of two conformers, which are stabilized by an intramolecular hydrogen bond between the amino group and the phenyl ring. The observed conformations were examined in light of the Shallenberger‐Acree‐Kier molecular theory of sweet taste, and they align with the hypothesized criteria. Furthermore, the study illustrates how conformational relaxation can alter the equilibrium conformational distribution, resulting in the absence of certain conformers in the conformational landscape.

## Graphical abstract

We provide experimental evidences that support the old theories of sweetness using dulcine as the archetypal molecule by means of Laser Ablation Chirped Pulse Fourier Transform Microwave Spectroscopy (LA‐CP‐FTMW) technique. Eluciding the structure of dulcin, we can decipher if it meets the structural requirements that a sweet molecule need to have according to the postulated theories.

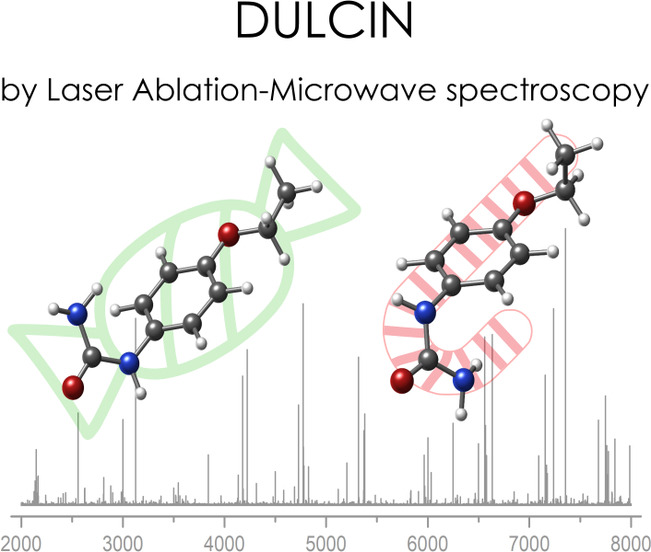

## Introduction

The taste perception is a complex process involving intricate chemical and biological interactions between ligands and specific proteins in taste receptor cells (TRCs). Five distinct taste types are associated with essential bodily functions: sweet, sour, salty, bitter, and umami.[^1^, ^2^] Sweetness is perceived by chemoreceptors within the taste buds that are capable of identifying specific molecular structures. These chemoreceptors are Type T1R2 and T1R3 taste receptors, which detect sweetness through the binding of sweet molecules.[^3^, ^4^, ^5^] Upon activation, these taste receptors initiate a signal transduction pathway that ultimately leads to the release of neurotransmitters in the brain.[^6^, ^7^, ^8^, ^9^, ^10^] Nevertheless, the binding sites of these receptors remain enigmatic, as there is limited information available on the structure of the receptor‐ligand complex. Consequently, the question of what renders a molecule sweet remains a mystery.

**Figure 1:**
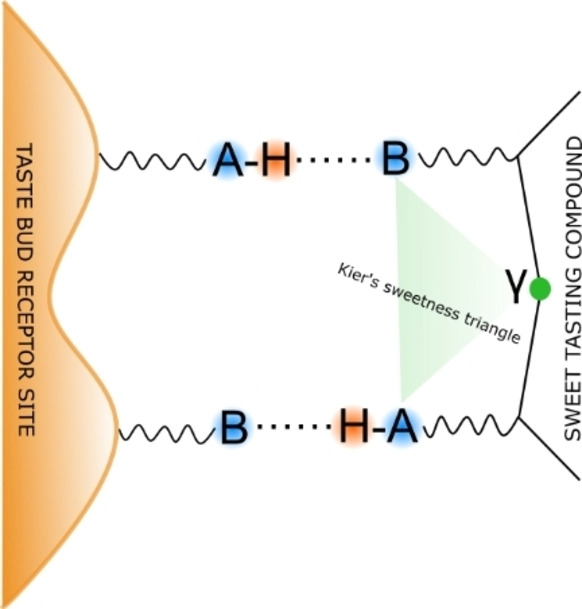
Sketch of the glucophore proposed by Shallenberger and Acree with the Kier contribution and its interaction via hydrogen bonding with the receptor site.

For more than a century, researchers have delved into the relationship between sweetness and the molecular structure of sweeteners. In 1963, R. S. Shallenberger put forth a molecular model for sweet taste, suggesting the existence of a fundamental molecular feature common to all sweet sugar molecules.[^11^, ^12^, ^13^, ^14^, ^15^, ^16^, ^17^] He suggested that a pair of functional groups could induce sweetness, one acting as a proton donor (AH) and the other as a proton acceptor (B), forming the glucophore (see Figure 1). A and B are electronegative atoms separated from 2.5 to 4 Å.^[18]^ The perception of sweetness arises from the interaction between the glucophore of a molecule and a corresponding glucophore pattern within the taste bud receptor site, facilitated by hydrogen bonds in an antiparallel manner. Subsequently, Kier introduced an updated theory that incorporated a third interacting site, referred to as γ, which amplifies the sweet flavor.[^19^, ^20^] This additional point interacts with the receptor through hydrophobic or van der Waals interactions. This three‐point contact AH/B/γ model is commonly referred to as “the sweetness triangle.”^[19]^ (see Figure 1). New theories regarding sweet properties have emerged,[^21^, ^22^, ^23^] all concurring on the necessity of a glucophore. However, determining the precise location of this glucophore requires thorough knowledge of the sweetener‘s structure. Unfortunately, most sweeteners are solids with high melting points, posing challenges for studying them in the gas phase. Consequently, structural data is primarily limited to condensed phases, where geometry is influenced by crystal packing forces and solvent effects, thereby obscuring detailed structural information essential for locating the glucophore in sweeteners. Consequently, conventional methodologies fall short in providing conclusive experimental evidence supporting potential structure‐property relationships of sweeteners.

In the past decade, the combination of laser ablation and high‐resolution Fourier Transform microwave techniques^[24]^ has facilitated the initial conformational characterization of D‐glucose and D‐galactose under the isolation conditions of supersonic expansion.[^25^, ^26^] his approach has emerged as a crucial tool for analyzing the structure of solid organic compounds.^[27]^ This experimental method has been applied to investigate the structure‐activity relationship of natural sweeteners, commencing with D‐Fructose and other ketohexoses^[28]^ utilizing laser ablation chirped pulse Fourier Transform Microwave (LA‐CP‐FTMW) spectroscopy. Furthermore, the scope of the study has extended to artificial sweeteners such as the sugar alcohols dulcitol and sorbitol,^[29]^ saccharin,^[30]^ and perillartine.^[31]^

Certain compounds containing a urea grouping exhibit a sweet taste and are typically classified as urea substances. Among them, phenyl ureas constitute the initial group of interest. Within this family, the position of the ring substituent alkoxy group significantly impacts the taste of the compound.^[32]^ Specifically, the ortho position renders the compound tasteless, the meta position imparts bitterness, while the para position elicits sweetness. An exemplar of a sweet‐tasting compound is p‐ethoxyphenylurea, commonly known as Dulcin (refer to Figure 1). Dulcin, an artificial sweetener belonging to the polyurea family, possesses a sweetness approximately 250 times greater than sucrose, rendering it an efficient sweetening agent. It was initially synthesized in 1884 by Joseph Berlinerblau through the reaction between chlorine cyanide and p‐phenetidine (p‐C_2_H_5_O‐C_6_H_4_‐NH_2_).^[33]^ Initially touted as an ideal sweetener for individuals with diabetes, dulcin faced a ban as a food additive in 1950 due to its adverse carcinogenic effects.[^20^, ^34^, ^35^] Nonetheless, it continues to be investigated as a model compound[^36^, ^37^, ^38^] owing to its unique properties that may be transferrable to the synthesis of other more consumption‐suited molecules. Despite studies on some derivatives of polyureas utilizing techniques such as X‐rays, NMR spectroscopy, or semi‐empirical calculations,^[39]^ an accurate structural determination for dulcin remains elusive. Consequently, precise data confirming the sweetness theory for this molecule are lacking.

In this report, we present the first rotational study of the artificial sweetener dulcin using our Laser Ablation Chirped Pulse Fourier Transform Microwave (LA‐CP‐FTMW) technique. As demonstrated, dulcin manifests four stable conformers, two of which are identified, while one remains elusive due to conformational interconversion. Notably, all conformers adhere to the prerequisites of sweetness theory, for which we provide structural information and identify potential glucophore sites. Our objective is to showcase our technique as the ultimate tool for discerning prospective sweet molecules and contributing to the exploration of novel and healthier artificial sweeteners.

## Results and Discussion

### Theoretical Modeling

To conduct gas phase experiments, we follow a procedure that integrates computational and spectroscopic methodologies. Computational techniques are used to predict the most stable conformers that are expected to be characterized under the isolation conditions of the supersonic expansion. These structures yield a set of theoretical rotational parameters, which serve as a guide for spectrum acquisition and analysis. With this information, we are ready to perform spectroscopic experiments.

**Figure 2:**
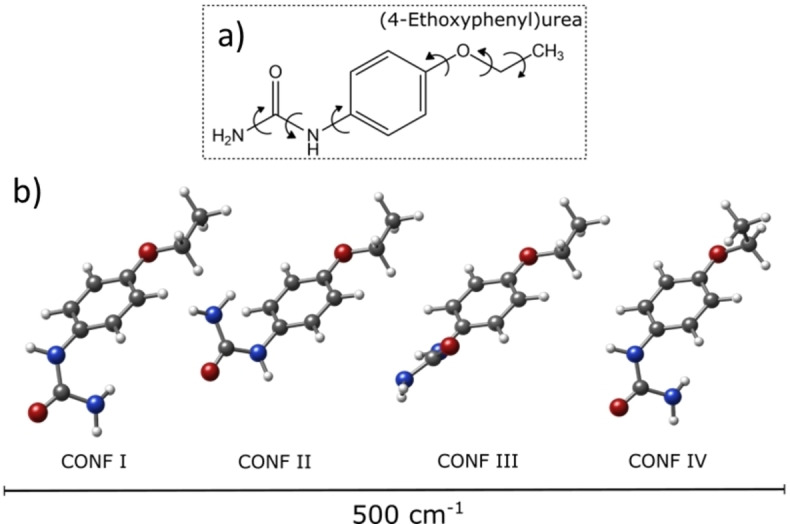
(a) The chemical structure of dulcin with its torsional degrees of freedom. (b) The four most stable conformers predicted in a 500 cm^−1^ energy window.

Dulcin molecule features six torsions (refer to Figure 2a), providing a diverse conformational landscape. In the first step, an automated search of the potential energy surface is conducted using fast molecular mechanics methods (MMFFs) to explore this landscape. Two search algorithms were employed – the “Large scales Low Mode” algorithm (that utilizes frequency modes to create new structures) and a Monte Carlo‐based search implemented in Macromodel.^[40]^ This advanced search process generated 17 potential conformations within a 25 kJ mol^−1^ energy window (see supplementary material).

The second step consists of a full geometrical optimization using the Gaussian 2016 suite of programs^[41]^ and, as starting geometries, the 17 previously obtained structures. The model of choice was the Møller–Plesset (MP2) perturbation theory in the frozen core approximation^[42]^ with Pople's 6‐311++G(d,p) basis set.^[43]^ Each conformer was confirmed to be a local minimum in the potential energy surface (PES) by checking that its Hessian matrix had no imaginary eigenvalue. Additionally, we optimized the structures using B3LYP density functionals, including the Grimme dispersions[^44^, ^45^] with Pople's 6‐311++G(d,p) basis set. The combination of both levels of theory has worked satisfactorily in previous studies of similar molecules.^[46]^ A total of 13 conformers were finally obtained. Their relative energy, rotational constants (*A*, *B*, *C*), and electric dipole moment components (*μ_a_*, *μ_b_*, and *μ_c_*) are summarized in Tables S1–S2 of the supplementary information. All the structures are asymmetric tops close to the prolate limit, with appreciable dipole moments in the three principal axes.

## Experimental Details

A commercial sample of dulcin (melting point 160 °C; Merck) was used without any additional purification. The compound‘s fine powder was mixed with a small amount of commercial binder to prepare a solid rod. This rod was placed in the ablation nozzle, and a picosecond Nd : YAG laser (12 mJ per pulse, 20 ps pulse width) was used to vaporize the mixture. The resulting laser ablation products were then supersonically expanded using a flow of neon gas (10 bar) and analyzed using Chirp Pulsed Fourier Transform Microwave (CP‐FTMW) spectroscopy. Chirped pulses of 4 μs directly generated by the 24 GS s^−1^ arbitrary waveform generator were amplified to about 300 W peak power using a traveling wave tube amplifier. The resulting pulses were transmitted and detected by broadband microwave horn antennas in a high‐vacuum chamber, interacting with the molecular supersonic expansion. At a repetition rate of 2 Hz, 115000 free induction decays (4 FID emissions per gas pulse), each with a 10 μs length, were averaged and digitized using a 50 GS s^−1^ digital oscilloscope. The frequency‐domain spectrum in the 2–8 GHz frequency range was obtained by taking a fast Fourier transform (FFT) following the application of a Kaiser–Bessel window to improve the baseline resolution.

### Rotational Spectrum and Conformational Analysis

The frequency range of 2–8 GHz was chosen to collect the spectrum of dulcin (see Figure 2 for an inset and Figure S1 for complete spectrum). Prior to spectrum analysis, lines related to photofragmentation and water clusters were eliminated from the spectrum. Guided by theoretical predictions of the spectra for the low‐energy conformers, an intense a‐type R‐branch progression of a near prolate asymmetric rotor^[47]^ separated about 600 MHz (approximately B+C values of rotational constants), corresponding to values of J ranging from J=3 to 12 was ascribed to a first rotamer 1. Through an iterative process of measurement and fitting,[^48^, ^49^] of μ_a_‐type and a few μ_c_‐type transitions, totaling 52, a set of experimental rotational constants was derived. The spectroscopic constants of rotamer 1 are collected in the first column of Table 1.

**Table 1:** Experimentally derived rotational parameters for dulcin rotamers, along with those theoretically calculated at MP2/6‐311++G (d,p) level. See the ESI for the calculated values of the rest of the conformers.

	Experimental		MP2/6‐311++G(d,p)			
	Rotamer 1	Rotamer 2	I	II	III	IV
A^[a]^	3052.1532(53)^[g]^	2413.98(76)	2787	3068	2832	2394
B	304.82621(33)	336.70893(42)	312	304	311	339
C	292.43629(32)	319.08422(42)	292	290	286	322
D_J_	0.00589(93)	0.0116(17)				
D_JK_	−0.128(17)	−0.135(84)				
*|μ*_a_ *|*	Observed	Observed	4.9	5.1	0.6	4.6
*|μ*_b_ *|*	–	–	0.9	0.8	1.4	1.2
*|μ*_c_ *|*	Observed	–	0.9	1.5	1.9	1.1
*σ* ^[b]^	9.1	6.3				
*N* ^[c]^	52	16				
*ΔE* ^[d]^			0	21	399	356
*ΔE_ZPE_* ^[e]^			0	6	374	414
*ΔG* ^[f]^			9	0	399	489

[a] *A*, *B*, and *C* represent the rotational constants (in MHz); *μ*_*a*,_ *μ_b_* and *μ_c_* are the electric dipole moment components (in D). [b] RMS deviation of the fit (in kHz). [c] Number of measured transitions. [d] Relative energies (in cm^−1^) with respect to the global minimum. [e] Relative energies (in cm^−1^) with respect to the global minimum, taking into account the zero‐point energy (ZPE). [f] Gibbs energies (in cm^−1^) calculated at 298 K. [g] Standard error in parentheses in units of the last digit.

After discarding all the lines corresponding to rotamer 1 from the spectrum, another weak *a*‐type R‐branch progression belonging to a second rotamer, labeled as 2, was identified. A total of 17 μ_a_‐type R‐branch transitions were measured and fitted. Although μ_b_ or μ_c_‐type transitions were predicted, they were not observed. The experimental rotational constant values for this rotamer are compiled in the second column of Table 1. All measured lines for both rotameric species are listed in Tables S3 and S4 of the Supporting Information. No ^14^N nuclear quadrupole hyperfine structure resulting from the presence of two ^14^N nuclei in Dulcin was observed in any of the measured transitions of the two observed rotamers. The resolution achieved with the broadband technique was insufficient to resolve the hyperfine structure.

The assignment of the observed rotamers to the low‐energy conformers of dulcin is based on the quantitative agreement between their experimental and theoretical rotational constant values. Table 1 juxtaposes the values for rotamers 1 and 2 with those predicted at the MP2/6‐311++G(d,p) level for the lower energy conformers depicted in Figure 1. The conformational identification was straightforward; there is excellent agreement between the experimental values of rotamer 1 and those calculated for conformer II. Furthermore, the observation of intense μ_a_‐type and weaker μ_c_‐type lines for rotamer 1 aligns with the predicted magnitudes of the dipole moment components for conformer II. As for rotamer 2, its rotational constants align with those predicted for conformer IV. The observation of the a‐type spectrum and the absence of detection of the μ_b_‐ and μ_c_‐type transitions also corresponds with the predicted values of the dipole moment components for conformer IV. Demonstrating the consistency of the overall assignment, a scale factor ranging from 0.990 to 1.008 reconciles the theoretical values for conformers II and IV with the experimental values for rotamers 1 and 2, respectively (Table 1).

### Conformational Relaxation

Based on predictions, four conformers within a 500 cm^−1^ energy window were expected to be present in the supersonic expansion. Surprisingly, only conformers II and IV have been experimentally detected. Even more surprising is the absence of conformer I, the global minimum in Table 1, in the supersonic expansion of our experiment. Conformer I is isoenergetic with conformer II and is an asymmetric rotor with a very high μa dipole moment component (see Table 1). Despite this, no evidence was found for the most intense a‐type R branch transitions. This discrepancy raises questions about the ab initio MP2/6‐311++G(d,p) predicted relative stabilities of the dulcine conformers, which appears to contradict the experimental results. Consequently, we extended our calculations on Dulcin and employed further DFT calculations. The energies were recalculated using the B3LYP‐GD3 methodology (see Table S6), and conformer II is now identified as the global minimum, consistent with the experiment. However, both ab initio and DFT approaches predict conformers I and II as isoenergetic, which should result in conformer I being highly populated.

**Figure 3:**
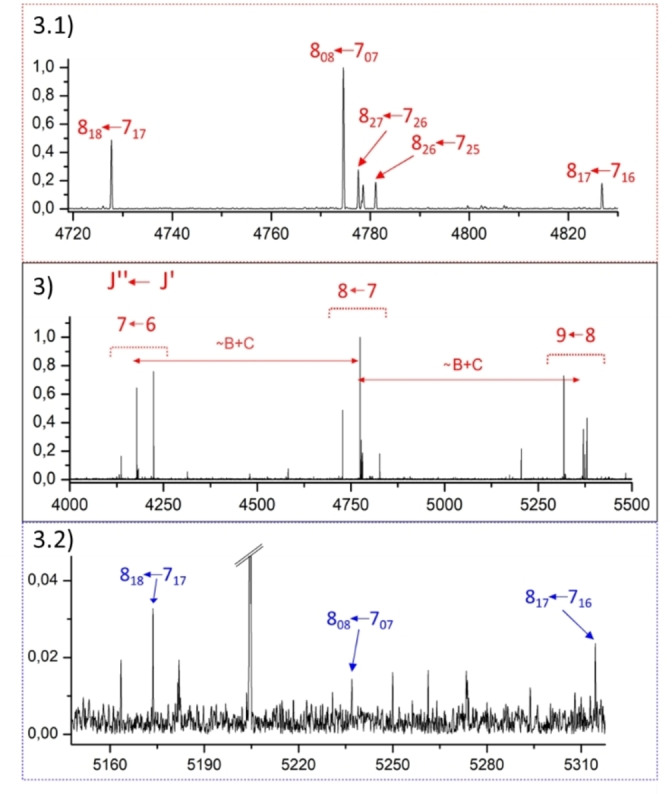
Section of the broadband rotational spectrum of dulcin from 4 to 5.5 GHz showing the *μ_a_*‐type R‐branch transitions (in red) separated approximately *B+C* value, corresponding to conformer II. 3.1) The upper section provides a zoomed‐in showing the *J*’’=8←*J*’=7 μ_*a*_‐type *R*‐branch transitions (in red) for conformer II. 3.2) The lower section provides a zoomed‐in showing the *J*’’=8←*J*’=7 μ_*a*_‐type *R*‐branch transitions (in blue) for conformer IV.

It is a known fact that a supersonic expansion can alter the conformational distribution during the cooling process, with certain conformers that are thermally populated at pre‐expansion conditions potentially relaxing to lower energy conformers through collisions. Consequently, these species may not be present after the subsequent jet expansion.^[50]^ This phenomenon typically occurs when the isomerization barrier is sufficiently low.^[51]^ Ruoff *et al*.^[52]^ have deduced that collisional removal of a higher energy conformer is possible when the isomerization barrier is lower than 400 cm^−1^. To investigate whether isomerization from conformer I to II occurs, the potential energy surface connecting both structures was explored by rotating the CCNC and NCNH torsional angles using the B3LYP‐GD3BJ method. As depicted in Figure 3, the computed interconversion barrier is shallow, approximately 140 cm^−1^, suggesting a significant population transfer from conformer I to conformer II, which likely renders conformer I undetectable.

Our findings indicate that conformer III does not exhibit any apparent interconversion barrier, as illustrated in Figure S2. While it may not have been detected, we attribute this to other contributing factors. Considering its energetics, the population in the jet should be comparable to that of conformer IV. However, its weak dipole moment is substantially lower than that of conformer IV, which already displayed weak rotational transitions. This suggests that the signal for conformer III is likely below the instrument‘s sensitivity threshold.

### Structure‐Sweetness Relationship

It has been observed that approximately 90 % of dulcin's abundance consists of conformers I and II, based on the relative intensities of rotational transitions and assuming complete conformational relaxation from conformer I to II. These observations are consistent with the predicted relative energies presented in Table 1. Although conformer I was not detected in the supersonic expansion due to its conformational relaxation, its significance cannot be overlooked. Therefore, the discussion will encompass both conformers.

**Figure 4:**
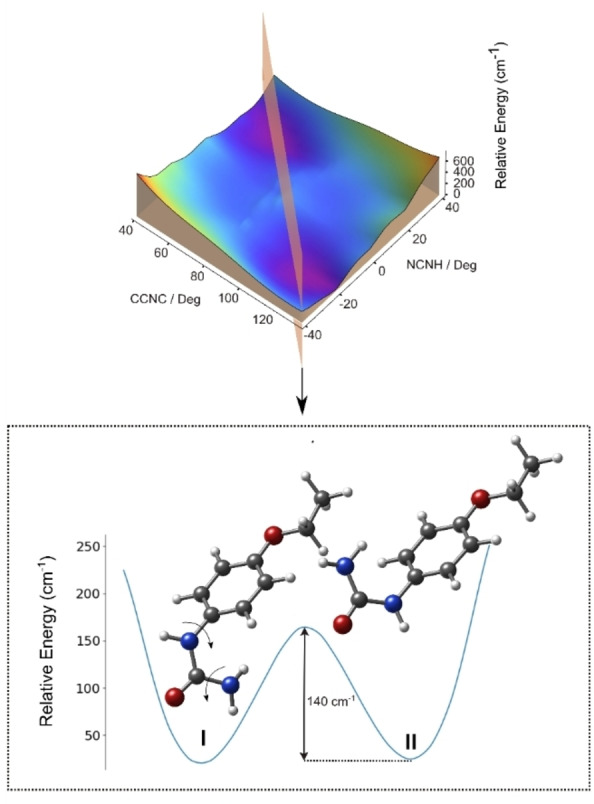
The potential energy surface (PES) of dulcin resulting from the rotation of the CCNC and NCNH torsional angles, calculated using the B3LYP‐GD3 method. The interconversion barrier is low, resulting in a relaxation of conformer I to II.

The most stable conformer II of this phenyl‐substituted urea adopts a favorable configuration wherein the NH_2_ group forms a hydrogen bond with the phenyl ring, positioning the ‐NH‐CO‐NH_2_ group at a right angle to the aromatic ring. This geometry is further influenced by the donor contribution of the alkoxy group (refer to Figure S3). The structure of the missed conformer I is analogous to conformer II, except that the urea group is positioned opposite to the alkoxy group. As depicted in Figure 4, the AH/B pair could correspond to the −CO−NH_2_ group, with the proton‐accepting species (B) being the −CO‐group and the NH_2_ group serving as the donor entity (AH) for both conformational species. In conformer II, the bonding of one hydrogen atom of the amino group to the phenyl ring activates the other. This argument strengthens the assignment of the terminal amino group to the AH sweetness function. The γ point, critical for stereo‐selectivity, could be the alkoxy group and is expected to distinguish between the two positions of the urea group. This contact point aligns with the proposal by Peer and coworkers.^[53]^

**Figure 5:**
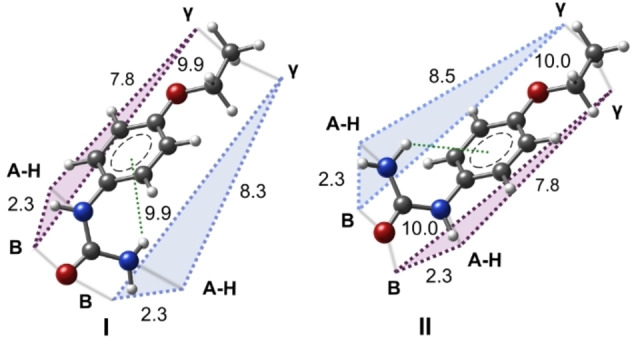
The two most stable conformers of Dulcin are presented. The sweetness triangle is outlined for each conformer, with the distances between the atoms (in Angstroms) indicated. Additionally, the interaction involving the NH_2_ group and the phenyl ring group is highlighted.

Moreover, a second glucophore unit could be proposed with the −NH−CO‐moiety of the molecule, where the −NH acts as a proton donor and the −CO‐group acts as a proton acceptor. As illustrated in Figure 5, the calculated distances for the three‐point contact AH/B/γ in all geometries align with those proposed by the sweetness theory. The two predominant structures of dulcin correspond to the sweetness theory proposed by Shallenberger–Acree–Kier.[^19^, ^54^] Due to the low interconversion barrier, these two structures should continuously interconvert at room temperature. This maximizes the probability of interaction with the receptor and could elucidate why this molecule exhibits such a high degree of sweetness. Our research provides valuable insights into the molecular structure of phenyl‐substituted urea and its stereo‐selectivity, which could be helpful in its applications.

## Conclusions

The benefits of vaporization by laser ablation and the high resolution and sensitivity achieved by rotational spectroscopy have led to groundbreaking insights into the structure of Dulcin, a common sweetener in the polyurea family. Among the four stable conformers that are thermally accessible in the equilibrium distribution, only two have been unequivocally identified by comparing experimental spectroscopic data with theoretical predictions. The most stable structure is stabilized by an intramolecular interaction between the NH_2_ group and the π electronic cloud of the phenyl ring group. However, due to collisional relaxation during the onset of the supersonic expansion, one of the predicted structures is “missing” due to conformational interconversion. The other conformer likely falls below the sensitivity threshold due to its low abundance and dipole moment. The two characterized forms, along with the postulated missing conformer, offer the first comprehensive understanding of the conformational behavior of Dulcin, which has remained unknown until now.

We have found that dulcin‘s different conformations fulfill the requirements of the sweetness theory. Utilizing the framework proposed by Shallenberger Acree–Kier, we have proposed a sweetness triangle for the two most stable conformations. We have observed a strong correspondence between the AH/B/γ three‐point contact, and the distances suggested by the sweetness theory. Furthermore, the interconversion between these dominant forms of dulcin indicates dynamic conformational behavior, potentially explaining its high sweetness. This revelation within the polyurea family has the potential to validate the sweetness theory and facilitate the development of new artificial sweeteners.

## Conflict of Interests

The authors declare no conflict of interest.

1

## Supporting information

As a service to our authors and readers, this journal provides supporting information supplied by the authors. Such materials are peer reviewed and may be re‐organized for online delivery, but are not copy‐edited or typeset. Technical support issues arising from supporting information (other than missing files) should be addressed to the authors.

Supporting Information

## Data Availability

The data that support the findings of this study are available in the supplementary material of this article.

## References

[1] R. Harper, *Human Senses in Action*, Edinburgh, Churchill Livingstone, **1972**.

[2] R. W. Moncrieff, *The Chemical Senses*, Leonard Hill, **1967**.

[3] G. Nelson , M. A. Hoon , J. Chandrashekar , Y. Zhang , N. J. P. Ryba , C. S. Zuker , Cell 2001, 106, 381–390. 11509186 10.1016/s0092-8674(01)00451-2

[4] R. F. Margolskee , J. Biol. Chem. 2002, 277, 1–4. 11696554 10.1074/jbc.R100054200

[5] X. Li , L. Staszewski , H. Xu , K. Durick , M. Zoller , E. Adler , Proc. Natl. Acad. Sci. USA 2002, 99, 4692–4696. 11917125 10.1073/pnas.072090199 PMC123709

[6] E. Oertly , R. G. Myers , J. Am. Chem. Soc. 1919, 41, 855–867.

[7] S. Kodama , Journal of the Tokyo Chemical Society 1920, 41, 495–534.

[8] Y. Tsuzuki , Science (Japan) 1947, 17, 175.

[9] K. Kaneko , Journal of chemical sociaty japan 1938, 59, 433–439.

[10] A. R. Lawrence , L. N. Ferguson , Nature 1959 183 : 4673 1959, 183, 1469–1471. 10.1038/1831469a0 13657176

[11] R. S. Shallenberger , Agric. Sci. Rev. 1964, 2.

[12] R. S. Shallenberger , T. E. Acree , Nature 1967 216 : 5114 1967, 216, 480–482. 10.1038/216480a0 6057249

[13] R. S. Shallenberger , T. E. Aeree , J. Agric. Food Chem. 1969, 17, 701–703.

[14] R. S. Shallenberger , T. E. Acree , C. Y. Lee , Nature 1969 221 : 5180 1969, 221, 555–556. 10.1038/221555a0 5794306

[15] R. S. Shallenberger , J. Food Sci. 1963, 28, 584–589.

[16] R. S. Shallenberger , T. E. Acree , W. E. Guild , J. Food Sci. 1965, 30, 560–563.

[17] T. E. Shallenberger , R. S. Acree , in Taste (Ed.: L. M. Beidler ), Springer Berlin Heidelberg, Berlin, Heidelberg, 1971, pp. 221–277.

[18] R. S. Shallenberger , Food Chem. 1996, 56, 209–214.

[19] L. B. Kier , J. Pharm. Sci. 1972, 61, 1394–1397. 5068944 10.1002/jps.2600610910

[20] A. van der Heijden , H. van der Wel , H. G. Peer , Chem. Senses 1985, 10, 73–88.

[21] C. Nofre , J.-M. Tinti , Food Chem. 1996, 56, 263–274.

[22] M. Mathlouthi , J. A. Kanters , G. G. G. Birch , G. E. Dubois , D. E. Walters , M. S. Kellog , in Sweet-Taste Chemoreception (Eds.: M. Mathlouthi , J. A. Kanters , G. G. Birch ), Elsevier Applied Science, Champagne-Ardenne., Université de Reims, 1993, p. 425.

[23] R. S. Shallenberger, *Taste Chemistry*, Springer US, **2012**.

[24] C. Cabezas , J. L. Alonso , J. C. López , S. Mata , J. C. Lõpez , S. Mata , Angew. Chem. Int. Ed. 2012, 51, 1375–1378. 10.1002/anie.201106621 22223259

[25] J. L. Alonso , M. A. Lozoya , I. Peña , J. C. López , C. Cabezas , S. Mata , S. Blanco , Chem. Sci. 2014, 5, 515–522.

[26] I. Peña , C. Cabezas , J. L. Alonso , Chem. Commun. 2015, 51, 10115. 10.1039/c5cc01783a 26008585

[27] J. L. Alonso , J. C. López , in Top Curr Chem (Eds.: R. A., O. J. ), Springer Verlag, 2015, pp. 335–402.

[28] C. Bermúdez , I. Peña , C. Cabezas , A. M. Daly , J. L. Alonso , ChemPhysChem 2013, 14, 893–895. 23401133 10.1002/cphc.201300057

[29] E. R. Alonso , I. León , L. Kolesniková , J. L. Alonso , ChemPhysChem 2018, 19, 3334–3340. 30370987 10.1002/cphc.201800977

[30] E. R. Alonso , I. León , L. Kolesniková , J. L. Alonso , J. Phys. Chem. A 2019, 123, 2756–2761. 30844277 10.1021/acs.jpca.8b12211

[31] G. Juárez , M. Sanz-Novo , J. L. Alonso , E. R. Alonso , I. León , Molecules 2022, 27, 1924. 35335289 10.3390/molecules27061924 PMC8954681

[32] R. W. Moncrieff, *The Chemical Senses*, Leonard Hill, **1967**.

[33] J. Berlinerblau , J. Prakt. Chem. 1884, 30, 97–115.

[34] R. S. Shallenberger , Taste Chemistry, Edition 1. London: Chapman & Hall, 1993.

[35] R. H. Goldsmith , J. Forensic Sci. 1986, 31, 11892 J. 3511177

[36] N. Frollof , E. Lloret , J.-M. Martinez , A. Faurion , Chem. Senses 1998, 23, 197–206. 9589167 10.1093/chemse/23.2.197

[37] G. Nelson , M. A. Hoon , J. Chandrashekar , Y. Zhang , N. J. P. Ryba , C. S. Zuker , Cell 2001, 106, 381–390. 11509186 10.1016/s0092-8674(01)00451-2

[38] Y. Uesawa , A. G. Staines , D. Lockley , K. Mohri , B. Burchell , Arch. Toxicol. 2007, 81, 163–168. 16897040 10.1007/s00204-006-0138-5

[39] M. R. Ciajolo , F. Lelj , T. Tancredi , P. A. Temussi , A. Tuzi , J. Med. Chem. 1983, 26, 1060–1065. 6864733 10.1021/jm00361a021

[40] Schrödinger INC., **2018**.

[41] G. A. Frisch, M. J. Trucks, G. W. Schlegel, H. B. Scuseria, G. E. Robb, M. A. Cheeseman, J. R. Scalmani, G. Barone, V. Petersson, *Gaussian, Inc*. **2016**.

[42] C. Møller , M. S. Plesset , Phys. Rev. 1934, 46, 618–622.

[43] M. J. Frisch , J. A. Pople , J. S. Binkley , J. Chem. Phys. 1984, 80, 3265–3269.

[44] A. D. Becke , J. Chem. Phys. 1993, 98, 1372–1377.

[45] S. Grimme , J. Antony , S. Ehrlich , H. Krieg , J. Chem. Phys. 2010, 132, 154104. 20423165 10.1063/1.3382344

[46] V. Cortijo , E. R. Alonso , S. Mata , J. L. Alonso , J. Phys. Chem. A 2018, 122, 646–651. 29215883 10.1021/acs.jpca.7b08882

[47] W. Gordy, R. L. Cook, *Microwave Molecular Spectra*, Wiley, **1984**.

[48] D. F. Plusquellic, “JB95 Spectral Fitting Program,” **n.d**.

[49] H. M. Pickett , J. Mol. Spectrosc. 1991, 148, 371–377.

[50] T. F. Miller , D. C. Clary , A. J. H. M. Meijer , J. Chem. Phys. 2005, 122, 244323. 16035773 10.1063/1.1927527

[51] C. Cabezas , M. Varela , J. L. Alonso , Angew. Chem. Int. Ed. 2017, 56, 6420–6425. 10.1002/anie.201702425 28455904

[52] R. S. Ruoff , T. D. Klots , T. Emilsson , H. S. Gutowsky , J. Chem. Phys. 1990, 93, 3142–3150.

[53] A. Van Der Heijden , H. Van Der Wel , H. G. Peer , Chem. Senses 1985, 10, 73–88.

[54] R. S. Shallenberger , T. E. Acree , Nature 1967 216 : 5114 1967, 216, 480–482. 10.1038/216480a0 6057249

