# Supplementary material for: Rotational Spectroscopy as a Tool to Characterize Sweet Taste: The Study of Dulcin
Source: ChemistryOpen. 2024 Jul 25;13(11):e202400159. doi: 10.1002/open.202400159 (PMC11564861; doi:10.1002/open.202400159)
Supplement: Supplementary file 1 — Supporting Information [file OPEN-13-e202400159-s001.pdf]

ChemistryOpen

Supporting Information

Rotational Spectroscopy as a Tool to Characterize Sweet Taste: The Study of Dulcin

Gabriela Juarez, Elena R. Alonso,* Raúl Aguado, and Iker León

Supplementary Information

for

Rotational Spectroscopy as a Tool to Characterize Sweet Taste: The Study of Dulcin

Gabriela Juarez, Raúl Aguado, Iker León, Elena R. Alonso*

Table S1- Theoretical spectroscopic constants for the three lowest energy conformers of dulcin calculated at the MP2/6-311++G(d,p) level of theory.

**MP2/6-311++G(d,p)**	**I**	**II**	**III**
***A* / MHz** [a]	2787.4	3067.9	2831.6
***B* / MHz**	312.0	304.4	311.0
***C* / MHz**	292.6	290.3	286.0
μ_a/D [b]	4.9	5.1	0.6
μ_b/D	0.9	0.8	1.4
μ_c/D	0.9	1.5	1.9
$\chi_{aa(-NH_2)}$ /MHz [c]	2.9	2.8	2.5
$\chi_{bb(-NH_2)}$ /MHz	0.0	-0.3	1.8
$\chi_{cc(-NH_2)}$ /MHz	-2.9	-2.5	-4.3
$\chi_{aa(-NH-)}$ /MHz	1.7	1.9	2.4
$\chi_{bb(-NH-)}$ /MHz	-1.1	-1.6	-0.4
$\chi_{cc(-NH-)}$ /MHz	-0.7	-0.3	-2.0
ΔE/cm^{-1} [d]	0	21	399
ΔE_{zpe}/cm^{-1} [e]	0	6	374
ΔG/cm^{-1} [f]	9	0	399

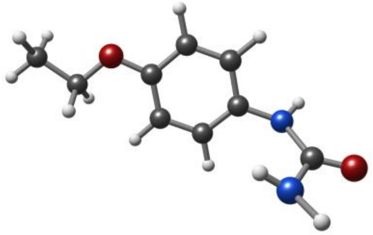

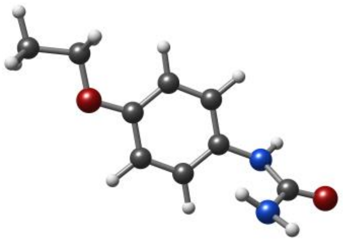

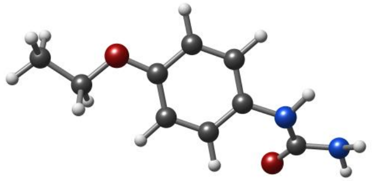

[a] *A*, *B*, and *C* represent the rotational constants (in MHz); μ_a, μ_b and μ_c are the electric dipole moment components (in D). [b] RMS deviation of the fit (in kHz). [c] χ_{aa}, χ_{bb}, χ_{cc} are the diagonal elements of the ^{14}N nuclear quadrupole coupling tensor [d] Relative energies (in cm^{-1}) with respect to the global minimum. [e] Relative energies (in cm^{-1}) with respect to the global minimum, taking into account the zero-point energy (ZPE). [f] Gibbs energies (in cm^{-1}) calculated at 298 K.

Continuation

MP2/6-311++G(d,p)	IV	V	VI
A / MHz	2393.7	3207.6	2122.5
B / MHz	339.1	302.2	357.0
C / MHz	321.7	283.6	325.6
μ_a/D	4.6	0.0	5.0
μ_b/D	1.2	3.7	0.4
μ_c/D	1.1	1.6	1.0
$\chi_{aa(-NH_2)}$ /MHz	2.7	2.4	2.8
$\chi_{bb(-NH_2)}$ /MHz	1.1	1.7	-4.1
$\chi_{cc(-NH_2)}$ /MHz	-3.8	-4.1	1.3
$\chi_{aa(-NH-)}$ /MHz	1.4	2.4	1.9
$\chi_{bb(-NH-)}$ /MHz	0.2	-1.0	-4.2
$\chi_{cc(-NH-)}$ /MHz	-1.5	-1.5	2.3
ΔE/cm^{-1}	356	452	399
ΔE_{zpe}/cm^{-1}	414	421	438
ΔG/cm^{-1}	489	442	497
	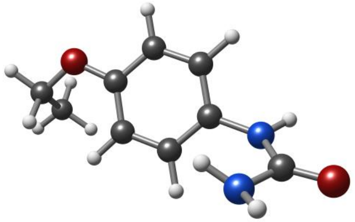	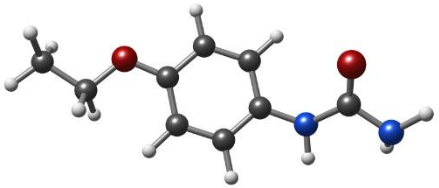	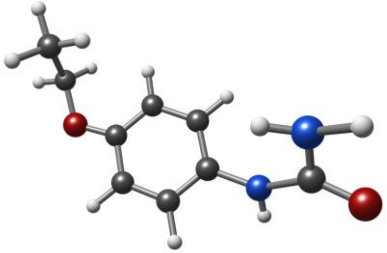

Continuation

MP2/6-311++G(d,p)	VII	VIII	IX
A / MHz	2690.9	2321.3	2143.1
B / MHz	332.3	336.6	356.0
C / MHz	313.4	329.2	321.5
μ_a/D	5.0	5.2	1.0
μ_b/D	0.5	0.2	1.4
μ_c/D	1.5	1.3	1.0
$\chi_{aa(-NH_2)}$ /MHz	2.8	2.4	2.4
$\chi_{bb(-NH_2)}$ /MHz	0.7	1.5	2.2
$\chi_{cc(-NH_2)}$ /MHz	-3.6	-3.9	-4.6
$\chi_{aa(-NH-)}$ /MHz	1.9	1.9	2.5
$\chi_{bb(-NH-)}$ /MHz	-0.5	0.7	0.9
$\chi_{cc(-NH-)}$ /MHz	-1.4	-2.6	-3.3
ΔE/cm^{-1}	386	394	733
ΔE_{zpe}/cm^{-1}	442	450	786
ΔG/cm^{-1}	530	534	875

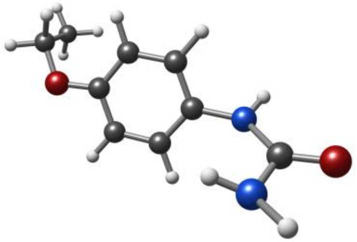

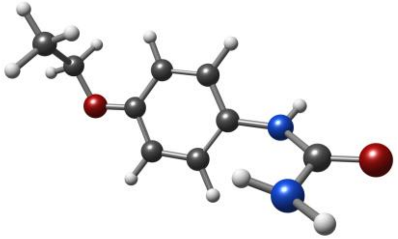

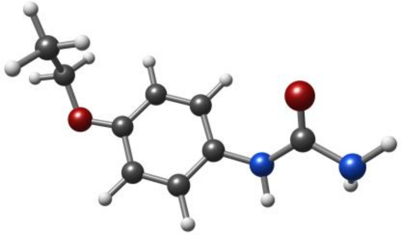

Continuation

MP2/6-311++G(d,p)	X	XI	XII
A / MHz	2361.1	2725.8	2172.5
B / MHz	340.7	311.5	351.6
C / MHz	315.4	295.1	335.3
μ_a/D	0.4	1.4	1.3
μ_b/D	1.0	2.6	2.8
μ_c/D	2.8	0.2	1.1
$\chi_{aa(-NH_2)}$ /MHz	2.50	2.27	2.2
$\chi_{bb(-NH_2)}$ /MHz	1.08	2.10	2.0
$\chi_{cc(-NH_2)}$ /MHz	-3.58	-4.38	-4.1
$\chi_{aa(-NH-)}$ /MHz	2.2	2.5	2.5
$\chi_{bb(-NH-)}$ /MHz	-1.6	0.3	1.4
$\chi_{cc(-NH-)}$ /MHz	-0.6	-2.8	-3.9
ΔE/cm^{-1}	752	888	850
ΔE_{zpe}/cm^{-1}	805	882	909
ΔG/cm^{-1}	916	446	895
	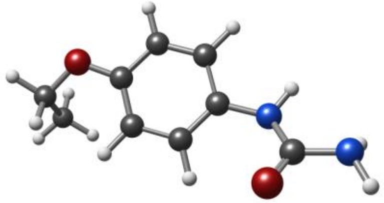	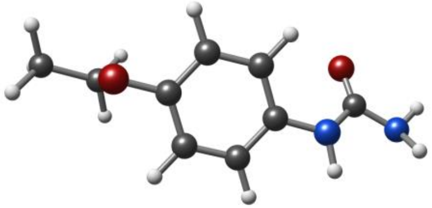	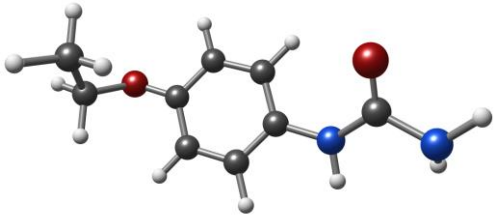

Continuation

MP2/6-311++G(d,p)	XIII
A / MHz	2584.3
B / MHz	334.5
C / MHz	319.8
μ_a/D	0.3
μ_b/D	3.9
μ_c/D	2.7
$\chi_{aa(-NH_2)}$ /MHz	2.5
$\chi_{bb(-NH_2)}$ /MHz	2.0
$\chi_{cc(-NH_2)}$ /MHz	-4.5
$\chi_{aa(-NH-)}$ /MHz	2.3
$\chi_{bb(-NH-)}$ /MHz	-0.2
$\chi_{cc(-NH-)}$ /MHz	-2.1
ΔE/cm^{-1}	924
ΔE_{zpe}/cm^{-1}	964
ΔG/cm^{-1}	807

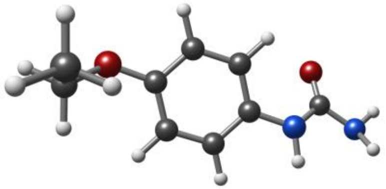

Table S2. Theoretical spectroscopic constants for the three lowest energy conformers of dulcin calculated at the B3LYP/6-311++G(d,p) level of theory including the Grimme dispersion. Conformers A and B are new conformers that do not appear in the MP2 optimization.

B3LYP/6-311++G(d,p)	II	I	III
A / MHz[a]	3112.6	2813.8	2893.15
B / MHz	302.5	310.1	307.01
C / MHz	288.1	290.0	278.74
μ_a/D[b]	5.4	5.1	0.5
μ_b/D	0.5	1.2	2.3
μ_c/D	1.5	1.0	-1.1
$\chi_{aa(-NH_2)}$ /MHz[c]	2.9	3.0	2.5
$\chi_{bb(-NH_2)}$ /MHz	-0.2	0.3	-4.9
$\chi_{cc(-NH_2)}$ /MHz	-2.8	-3.2	-4.9
$\chi_{aa(-NH-)}$ /MHz	2.1	1.9	2.5
$\chi_{bb(-NH-)}$ /MHz	-1.3	-0.8	1.6
$\chi_{cc(-NH-)}$ /MHz	-0.8	-1.2	-4.1
ΔE/cm^{-1} [d]	7.49	0	331
ΔE_{zpe}/cm^{-1}[e]	0	2.4	337
ΔG/cm^{-1}[f]	1.5	1.53	333

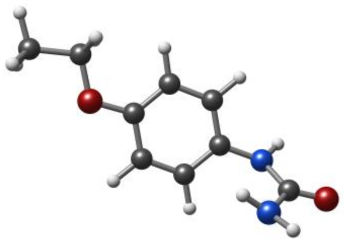
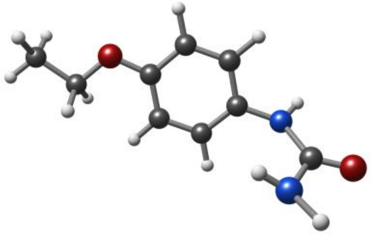
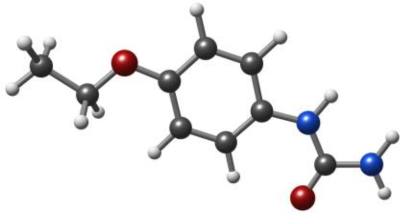

[a] *A*, *B*, and *C* represent the rotational constants; μ_a, μ_b and μ_c are the electric dipole moment components. [b] RMS deviation of the fit (in kHz). [c] χ_{aa}, χ_{bb}, χ_{cc} are the diagonal elements of the ^{14}N nuclear quadrupole coupling tensor [d] Relative energies with respect to the global minimum. [e] Relative energies with respect to the global minimum, taking into account the zero-point energy (ZPE). [f] Gibbs energies calculated at 298 K.

Continuation

B3LYP/6-311++G(d,p)	V	IV	VIII
A / MHz	3384.0	2386.5	2432.0
B / MHz	297.3	336.0	329.6
C / MHz	274.6	315.8	319.8
μ_a/D	0.3	5.1	5.4
μ_b/D	4.6	0.9	0.7
μ_c/D	1.0	1.3	1.3
$\chi_{aa(-NH_2)}$ /MHz	2.5	2.9	2.8
$\chi_{bb(-NH_2)}$ /MHz	2.4	-1.4	-2.1
$\chi_{cc(-NH_2)}$ /MHz	-4.9	-1.5	-0.7
$\chi_{aa(-NH-)}$ /MHz	2.5	1.7	2.1
$\chi_{bb(-NH-)}$ /MHz	1.5	-2.3	-3.2
$\chi_{cc(-NH-)}$ /MHz	-3.9	0.6	1.0
ΔE/cm^{-1}	419	339	354
ΔE_{zpe}/cm^{-1}	424	375	388
ΔG/cm^{-1}	378	391	415
	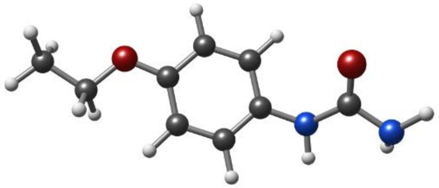	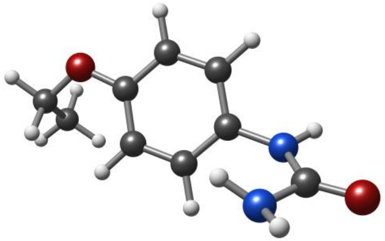	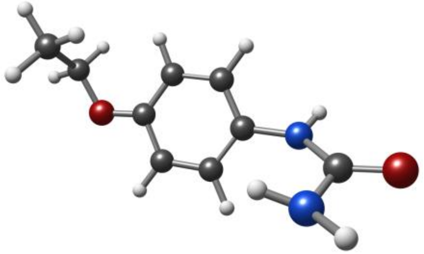

Continuation

B3LYP/6-311++G(d,p)	A	VI	X
A / MHz	2752.1	2166.9	2291.3
B / MHz	326.6	350.0	339.7
C / MHz	307.3	318.6	305.5
μ_a/D	5.3	5.2	0.6
μ_b/D	0.1	1.3	1.9
μ_c/D	1.6	0.7	1.8
$\chi_{aa(-NH_2)}$ /MHz	2.9	3.0	2.5
$\chi_{bb(-NH_2)}$ /MHz	0.7	1.3	2.2
$\chi_{cc(-NH_2)}$ /MHz	-3.7	-4.2	-4.7
$\chi_{aa(-NH-)}$ /MHz	2.1	1.9	2.3
$\chi_{bb(-NH-)}$ /MHz	-0.4	0.4	0.8
$\chi_{cc(-NH-)}$ /MHz	-1.7	-2.3	-3.1
ΔE/cm^{-1}	360	366	662
ΔE_{zpe}/cm^{-1}	389	398	709
ΔG/cm^{-1}	405	415	722
	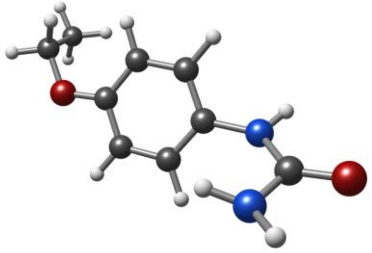	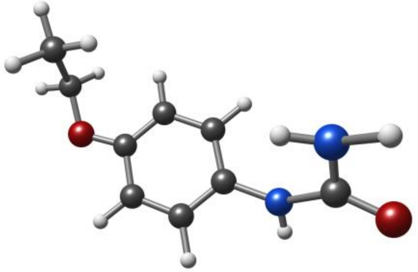	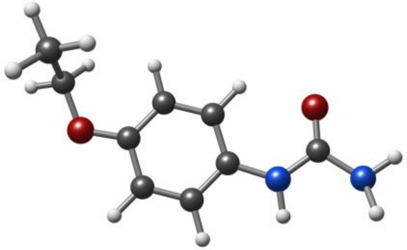

Continuation

B3LYP/6-311++G(d,p)	IX	B	XIII
A / MHz	2273.2	2747.5	2805.7
B / MHz	341.4	322.3	321.6
C / MHz	305.6	299.6	297.1
μ_a/D	0.7	0.4	0.6
μ_b/D	2.2	4.6	4.9
μ_c/D	0.3	1.4	0.6
$\chi_{aa(-NH_2)}$ /MHz	2.5	2.5	2.5
$\chi_{bb(-NH_2)}$ /MHz	2.1	2.2	2.2
$\chi_{cc(-NH_2)}$ /MHz	-4.5	4.7	4.7
$\chi_{aa(-NH-)}$ /MHz	2.6	2.5	2.3
$\chi_{bb(-NH-)}$ /MHz	2.1	0.7	2.1
$\chi_{cc(-NH-)}$ /MHz	-4.7	3.2	4.4
ΔE/cm^{-1}	658	774	786
ΔE_{zpe}/cm^{-1}	709	818	830
ΔG/cm^{-1}	721	788	819
	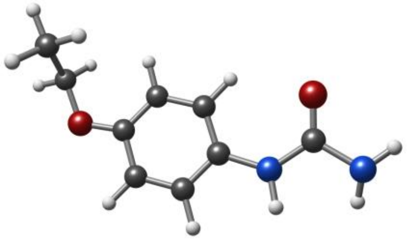	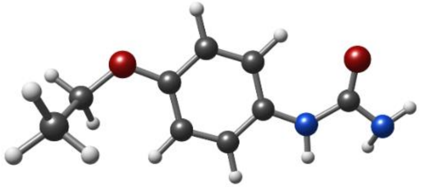	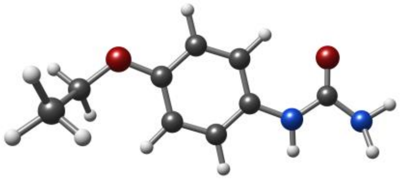

Table S3. Observed frequencies for rotational transitions of rotamer I (in MHz).

J'	K'_{-1}	K'_{+1}	J''	K''_{-1}	K''_{+1}	ν_{obs}	$\nu_{obs} - \nu_{cal}$
4	0	4	3	0	3	2388.6141	-0.0163
5	0	5	4	0	4	2985.4639	-0.0095
7	0	7	6	0	6	4178.4945	0.0050
8	0	8	7	0	7	4774.5844	0.0047
9	0	9	8	0	8	5370.3441	0.0071
10	0	10	9	0	9	5965.7245	0.0030
11	0	11	10	0	10	6560.6936	-0.0009
12	0	12	11	0	11	7155.2130	-0.0062
13	0	13	12	0	12	7749.2570	-0.0033
5	1	5	4	1	4	2955.1432	-0.0107
6	1	6	5	1	5	3546.0749	0.0053
7	1	7	6	1	6	4136.9328	0.0096
8	1	8	7	1	7	4727.7131	0.0079
9	1	9	8	1	8	5318.4118	0.0059
10	1	10	9	1	9	5909.0203	0.0037
11	1	11	10	1	10	6499.5305	0.0020
12	1	12	11	1	11	7089.9338	0.0003
13	1	13	12	1	12	7680.2025	-0.0210
6	1	5	5	1	4	3620.3992	-0.0050
7	1	6	6	1	5	4223.6394	-0.0021
8	1	7	7	1	6	4826.7912	-0.0111
9	1	8	8	1	7	5429.8910	0.0165
10	1	9	9	1	8	6032.8531	0.0071
11	1	10	10	1	9	6635.7082	0.0039
12	1	11	11	1	10	7238.4366	0.0005
13	1	12	12	1	11	7841.0248	-0.0032
8	2	7	7	2	6	4777.5711	-0.0140
8	2	6	7	2	5	4781.0779	-0.0153
9	2	8	8	2	7	5374.6062	0.0042
9	2	7	8	2	6	5379.6106	0.0004
10	2	9	9	2	8	5971.5601	0.0052
10	2	8	9	2	7	5978.4395	0.0049
11	2	10	10	2	9	6568.4416	0.0049
11	2	9	10	2	8	6577.5985	0.0007
12	2	11	11	2	10	7165.2515	0.0110
12	2	10	11	2	9	7177.1333	0.0038
13	2	12	12	2	11	7761.9600	0.0010
13	2	11	12	2	10	7777.0516	-0.0059
11	3	9	10	3	8	6571.1016	-0.0164
11	3	8	10	3	7	6571.1016	-0.0164
12	3	10	11	3	9	7168.7283	0.0018
12	3	9	11	3	8	7168.7283	0.0018
13	3	11	12	3	10	7766.2849	0.0207
13	3	10	12	3	9	7766.5188	-0.0098
12	4	9	11	4	8	7168.0385	-0.0065
12	4	8	11	4	7	7168.0385	-0.0065
13	4	10	12	4	9	7765.5309	0.0055
13	4	9	12	4	8	7765.5309	0.0055

2	1	1	1	0	1	3966.6454	0.0129
3	1	2	2	0	2	4582.5039	0.0081
4	1	3	3	0	3	5204.6043	-0.0173
5	1	4	4	0	4	5833.0894	-0.0036

Table S4. Observed frequencies for rotational transitions of rotamer II (in MHz).

J'	K'_{-1}	K'_{+1}	J''	K''_{-1}	K''_{+1}	ν_{obs}	$\nu_{obs} - \nu_{cal}$
8	0	8	7	0	7	5236.9654	-0.0059
10	0	10	9	0	9	6539.6407	0.0075
12	0	12	11	0	11	7838.1278	-0.0023
7	1	7	6	1	6	4527.4146	-0.0008
8	1	8	7	1	7	5173.6361	-0.0121
10	1	10	9	1	9	6465.4801	0.0093
11	1	11	10	1	10	7111.0177	-0.0033
12	1	12	11	1	11	7756.3118	0.0038
7	1	6	6	1	5	4650.7391	0.0070
8	1	7	7	1	6	5314.5387	0.0048
10	1	9	9	1	8	6641.3614	-0.0063
11	1	10	10	1	9	7304.3274	0.0023
12	1	11	11	1	10	7966.9183	-0.0041
10	2	9	9	2	8	6555.0779	-0.0589
11	2	10	10	2	9	7209.8486	-0.0742
12	2	11	11	2	10	7864.4295	-0.0720

Figure S1. Spectrum from 2-8GHz frequency range of dulcin obtained by means of CP-FTMW spectrometer.

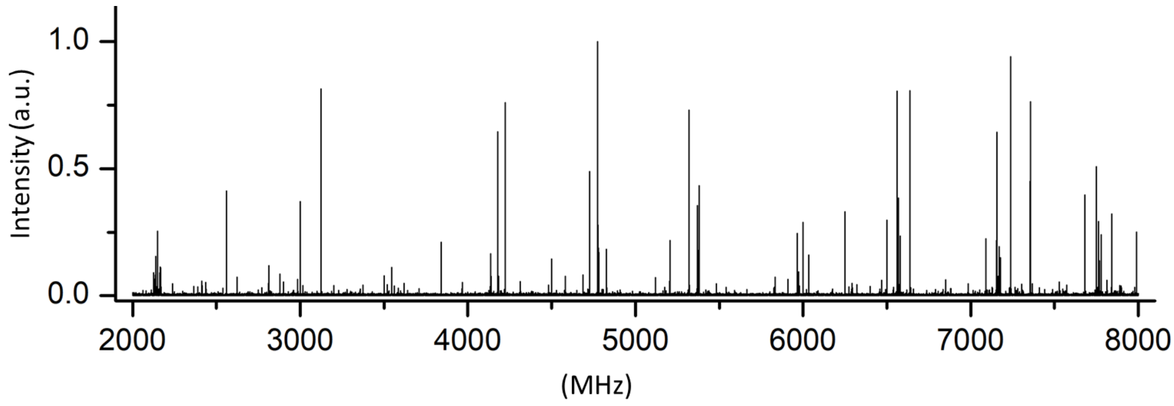

Figure S2. The potential energy surface of the interconversion between the conformers I and III of dulcin by rotating the CNCO torsional angle calculated at the B3LYP/6-311++G(d,p) level including Grimme dispersion. As can be seen, the calculated interconversion barrier is very high, so the explanation for the non-detection of conformer III must be due to other facts.

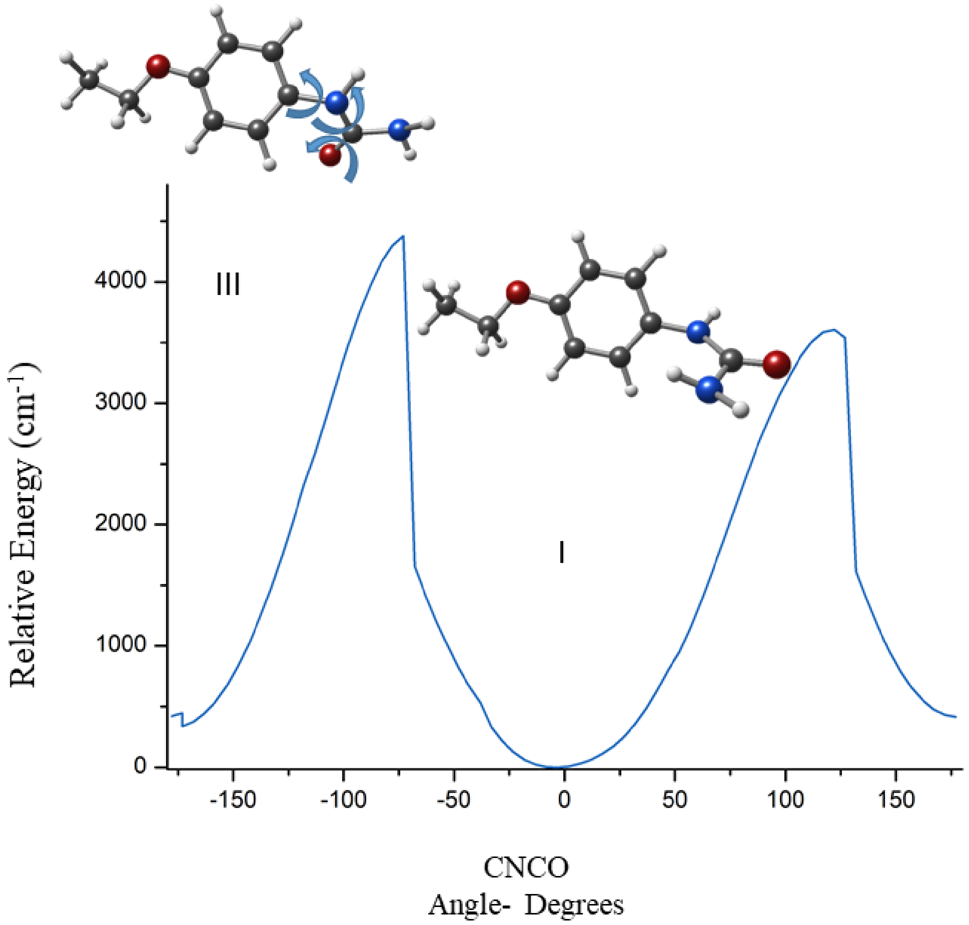

Figure S3. Non-covalent interactions of predicted conformers II and IV, corresponding to the characterized rotamers. The red color corresponds to the repulsion forces, the blue to the attraction forces and the green to weak interactions.

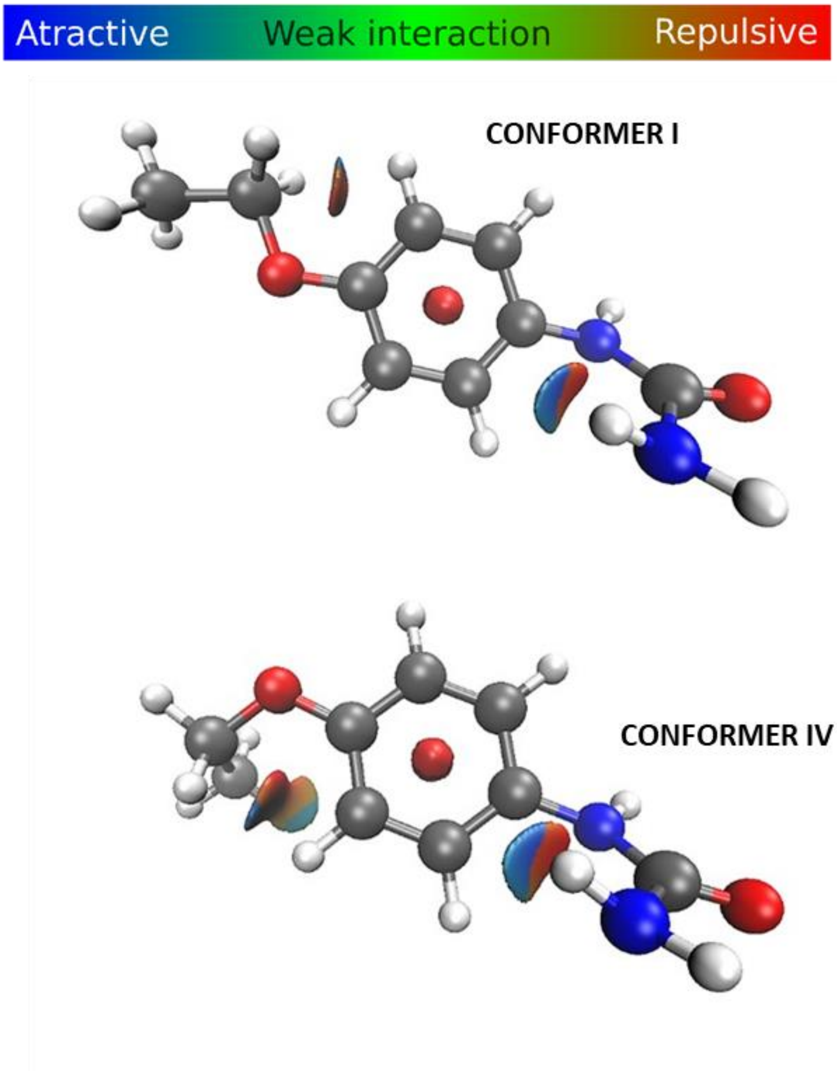

Table S5. Cartesian coordinates for conformer I of dulcin at the MP2/6-311++G(d,p) level.

Standard orientation

Center Number	Atomic Number	X	Y	Z
1	6	0.482338000	-0.689766000	-0.667053000
2	6	-0.905177000	-0.752588000	-0.476959000
3	6	1.201607000	0.460784000	-0.322134000
4	1	1.012924000	-1.543428000	-1.079399000
5	6	-1.593913000	0.369388000	0.009826000
6	1	-1.429820000	-1.661629000	-0.747834000
7	6	-0.878081000	1.535934000	0.327839000
8	8	-2.940635000	0.428228000	0.214640000
9	6	0.505690000	1.577395000	0.173906000
10	1	-1.426698000	2.394194000	0.704995000
11	1	1.058181000	2.474003000	0.444851000
12	7	2.604136000	0.520450000	-0.516401000
13	6	-3.698854000	-0.734891000	-0.116670000
14	1	-3.565739000	-0.970337000	-1.180495000
15	1	-3.344196000	-1.587357000	0.477220000
16	6	-5.149112000	-0.426548000	0.193020000
17	1	-5.770860000	-1.294342000	-0.045654000
18	1	-5.270054000	-0.189874000	1.252435000
19	1	-5.492609000	0.423963000	-0.399940000
20	1	2.997409000	1.344183000	-0.950750000
21	6	3.561835000	-0.274126000	0.096492000
22	8	4.756826000	-0.084857000	-0.074303000
23	7	3.058464000	-1.321348000	0.853695000
24	1	3.759833000	-1.721643000	1.459970000
25	1	2.145904000	-1.201901000	1.271231000

Table S6. Cartesian coordinates for conformer II of dulcin at the MP2/6-311++G(d,p) level.

Standard orientation

Center Number	Atomic Number	X	Y	Z
1	6	0.666241000	-0.952113000	-0.688243000
2	6	-0.695072000	-1.212163000	-0.550987000
3	6	1.179499000	0.321245000	-0.381211000
4	1	1.343070000	-1.730190000	-1.029694000
5	6	-1.580216000	-0.194726000	-0.154685000
6	1	-1.103531000	-2.191912000	-0.781830000
7	6	-1.079925000	1.086334000	0.123429000
8	8	-2.893168000	-0.553016000	-0.072144000

9	6	0.296699000	1.331086000	0.017931000
10	1	-1.734856000	1.893786000	0.429910000
11	1	0.688279000	2.316913000	0.258008000
12	7	2.562349000	0.594234000	-0.529828000
13	6	-3.823028000	0.469033000	0.285380000
14	1	-3.576270000	0.860323000	1.280956000
15	1	-3.760894000	1.291605000	-0.438886000
16	6	-5.202033000	-0.157466000	0.277295000
17	1	-5.950496000	0.592562000	0.549134000
18	1	-5.438818000	-0.544294000	-0.716293000
19	1	-5.251649000	-0.978046000	0.996458000
20	1	2.838163000	1.450330000	-0.991131000
21	6	3.602451000	0.003612000	0.173635000
22	8	4.758284000	0.377836000	0.043489000
23	7	3.235345000	-1.069347000	0.971066000
24	1	2.294904000	-1.081777000	1.340671000
25	1	3.959617000	-1.321109000	1.628008000

Table S7. Cartesian coordinates for conformer III of dulcin at the MP2/6-311++G(d,p) level.

Standard orientation

Center Number	Atomic Number	X	Y	Z
1	6	-0.476198000	-0.740548000	-0.436542000
2	6	0.919193000	-0.784787000	-0.310125000
3	6	-1.167625000	0.454170000	-0.211912000
4	1	-1.021680000	-1.642790000	-0.685654000
5	6	1.638687000	0.377345000	0.003760000
6	1	1.423006000	-1.729824000	-0.477339000
7	6	0.945252000	1.580274000	0.207375000
8	8	2.997201000	0.444230000	0.138792000
9	6	-0.443624000	1.614790000	0.108480000
10	1	1.511287000	2.473697000	0.454278000
11	1	-0.971376000	2.549538000	0.289083000
12	7	-2.573041000	0.542211000	-0.365377000
13	6	3.725171000	-0.763876000	-0.068285000
14	1	3.387803000	-1.526010000	0.646310000
15	1	3.543253000	-1.135372000	-1.085356000
16	6	5.191785000	-0.443683000	0.136159000
17	1	5.792422000	-1.345810000	-0.013031000
18	1	5.518567000	0.316551000	-0.576921000
19	1	5.362455000	-0.073026000	1.149393000
20	1	-2.936472000	1.485806000	-0.392368000
21	6	-3.452603000	-0.412765000	0.135245000
22	8	-3.115582000	-1.483099000	0.607503000
23	7	-4.782408000	0.006301000	0.059157000
24	1	-5.425868000	-0.755489000	0.223683000
25	1	-5.028431000	0.568574000	-0.743838000

Table S8. Cartesian coordinates for conformer IV of dulcin at the MP2/6-311++G(d,p) level.

Standard orientation

Center Number	Atomic Number	X	Y	Z
1	6	-1.063880000	0.398707000	0.328788000
2	6	-0.321756000	-0.789690000	0.326137000
3	6	1.034160000	-0.787569000	-0.025531000
4	1	1.678349000	0.423899000	-0.323545000
5	6	0.943376000	1.620221000	-0.288943000
6	1	-0.416286000	1.606419000	0.017547000
7	6	-0.811378000	-1.721364000	0.595634000
8	8	1.573412000	-1.728180000	-0.027491000
9	6	1.455719000	2.548648000	-0.524367000
10	1	-0.985161000	2.533237000	0.016667000
11	1	-2.766322000	1.099270000	1.332891000
12	7	-2.433787000	0.393061000	0.690589000
13	6	-3.464143000	-0.261901000	0.031603000
14	1	-4.629408000	-0.131619000	0.375218000
15	1	-2.203400000	-0.888399000	-1.472679000
16	6	-3.062240000	-1.110988000	-0.988574000
17	1	-3.832925000	-1.380037000	-1.583078000
18	1	2.986998000	0.546820000	-0.693964000
19	1	3.855050000	-0.563061000	-0.444754000
20	1	3.445663000	-1.473206000	-0.899070000
21	6	4.769907000	-0.307496000	-0.982770000
22	8	4.139798000	-0.747223000	1.038009000
23	7	4.854641000	-1.564001000	1.179303000
24	1	4.571888000	0.169077000	1.448006000
25	1	3.231416000	-0.982485000	1.596952000

Table S9. Cartesian coordinates for conformer V of dulcin at the MP2/6-311++G(d,p) level.

Standard orientation

Center Number	Atomic Number	X	Y	Z
1	6	-0.242160000	1.348080000	0.010667000
2	6	1.137067000	1.103452000	0.079228000
3	6	-1.144820000	0.317067000	-0.266427000
4	1	-0.611723000	2.356490000	0.187757000
5	6	1.618062000	-0.197787000	-0.120710000
6	1	1.805848000	1.928754000	0.294349000
7	6	0.709509000	-1.233601000	-0.393175000
8	8	2.934698000	-0.560456000	-0.069640000

9	6	-0.656713000	-0.981983000	-0.485540000
10	1	1.100148000	-2.234879000	-0.550314000
11	1	-1.345653000	-1.789205000	-0.704742000
12	7	-2.524181000	0.615246000	-0.395576000
13	6	3.875917000	0.470832000	0.214855000
14	1	3.810106000	1.253789000	-0.552377000
15	1	3.648446000	0.919800000	1.190803000
16	6	5.250804000	-0.165551000	0.220036000
17	1	6.009699000	0.592568000	0.435037000
18	1	5.306732000	-0.943796000	0.984421000
19	1	5.467130000	-0.611599000	-0.753303000
20	1	-2.739791000	1.600247000	-0.476860000
21	6	-3.516231000	-0.158009000	0.201433000
22	8	-3.320822000	-1.226515000	0.750655000
23	7	-4.772264000	0.446318000	0.122471000
24	1	-4.961470000	0.973407000	-0.718857000
25	1	-5.513983000	-0.195756000	0.365503000

Table S10. Cartesian coordinates for conformer VI of dulcin at the MP2/6-311++G(d,p) level.

Standard orientation

Center Number	Atomic Number	X	Y	Z
1	6	-1.103652000	0.590417000	-0.257524000
2	6	-0.340372000	-0.396605000	-0.893810000
3	6	1.056603000	-0.403635000	-0.787219000
4	1	1.709439000	0.616890000	-0.077689000
5	6	0.946491000	1.622740000	0.538317000
6	1	-0.444649000	1.606439000	0.455439000
7	6	-0.844411000	-1.166668000	-1.471205000
8	8	1.613652000	-1.185390000	-1.291057000
9	6	1.466585000	2.405708000	1.082704000
10	1	-1.029958000	2.375611000	0.953805000
11	1	-2.981717000	1.464118000	-0.590219000
12	7	-2.516520000	0.593850000	-0.370974000
13	6	-3.387871000	-0.381211000	0.091305000
14	1	-4.599983000	-0.243715000	0.022096000
15	1	-1.852395000	-1.442253000	0.963468000
16	6	-2.779246000	-1.531900000	0.570638000
17	1	-3.418093000	-2.104062000	1.103842000
18	1	3.062654000	0.746503000	0.048166000
19	1	3.878615000	-0.363733000	-0.336640000
20	1	3.632999000	-0.677170000	-1.358154000
21	6	4.890635000	0.045443000	-0.350382000
22	8	3.783260000	-1.517018000	0.650854000
23	7	2.774457000	-1.933545000	0.690850000
24	1	4.474571000	-2.313397000	0.357591000
25	1	4.055492000	-1.170058000	1.650819000

Table S11. Cartesian coordinates for conformer VII of dulcin at the MP2/6-311++G(d,p) level.

Standard orientation

Center Number	Atomic Number	X	Y	Z
1	6	-0.592566000	0.935793000	-0.732359000
2	6	0.730040000	1.328580000	-0.536069000
3	6	-1.035798000	-0.316290000	-0.271356000
4	1	-1.290959000	1.591777000	-1.244310000
5	6	1.648831000	0.465893000	0.084906000
6	1	1.084082000	2.294608000	-0.884679000
7	6	1.218995000	-0.796254000	0.522914000
8	8	2.910434000	0.964414000	0.242241000
9	6	-0.119512000	-1.173409000	0.350334000
10	1	1.899336000	-1.488386000	1.006077000
11	1	-0.456596000	-2.142514000	0.711197000
12	7	-2.375469000	-0.728061000	-0.483210000
13	6	3.953328000	0.042625000	0.573933000
14	1	4.800048000	0.683358000	0.827454000
15	1	3.685322000	-0.524664000	1.473185000
16	6	4.303411000	-0.870828000	-0.590853000
17	1	5.145827000	-1.513885000	-0.317169000
18	1	3.460864000	-1.506060000	-0.872639000
19	1	4.590262000	-0.270476000	-1.457832000
20	1	-2.543025000	-1.667682000	-0.816173000
21	6	-3.514462000	-0.136542000	0.045679000
22	8	-4.621734000	-0.630477000	-0.105537000
23	7	-3.301992000	1.072817000	0.688594000
24	1	-2.399595000	1.227720000	1.116224000
25	1	-4.096544000	1.354016000	1.244570000

Table S12. Cartesian coordinates for conformer VIII of dulcin at the MP2/6-311++G(d,p) level.

Standard orientation

Center Number	Atomic Number	X	Y	Z
1	6	-1.072679000	-0.162212000	-0.541526000
2	6	-0.619609000	1.168488000	-0.510301000
3	6	0.742632000	1.444077000	-0.408620000
4	1	1.685675000	0.402453000	-0.397138000
5	6	1.241425000	-0.927174000	-0.465161000
6	1	-0.131934000	-1.199357000	-0.522131000
7	6	-1.342385000	1.978125000	-0.557100000
8	8	1.104857000	2.467751000	-0.376995000
9	6	1.939171000	-1.756899000	-0.462940000
10	1	-0.475700000	-2.230949000	-0.546482000

11	1	-2.751678000	-1.157817000	-1.306093000
12	7	-2.456092000	-0.450278000	-0.647408000
13	6	-3.441523000	-0.132525000	0.276578000
14	1	-4.594186000	-0.514500000	0.140994000
15	1	-3.697149000	0.715174000	2.069367000
16	6	-3.030745000	0.693751000	1.311106000
17	1	-2.060460000	0.652896000	1.590406000
18	1	2.993071000	0.793750000	-0.356043000
19	1	3.978122000	-0.203206000	-0.069670000
20	1	3.874618000	-1.047007000	-0.762232000
21	6	4.925872000	0.291594000	-0.290313000
22	8	3.935018000	-0.651673000	1.383264000
23	7	2.986452000	-1.134047000	1.628943000
24	1	4.744877000	-1.362348000	1.576047000
25	1	4.065552000	0.211991000	2.040129000

Table S13. Cartesian coordinates for conformer IX of dulcin at the MP2/6-311++G(d,p) level.

Standard orientation

Center Number	Atomic Number	X	Y	Z
1	6	-0.337875000	-0.502404000	-0.737695000
2	6	1.062411000	-0.493901000	-0.727691000
3	6	-1.054249000	0.562365000	-0.179276000
4	1	-0.867638000	-1.346236000	-1.163494000
5	6	1.762276000	0.592813000	-0.182875000
6	1	1.586767000	-1.337273000	-1.163223000
7	6	1.042372000	1.666764000	0.360023000
8	8	3.125970000	0.719078000	-0.165152000
9	6	-0.351759000	1.650842000	0.361409000
10	1	1.592946000	2.503422000	0.780198000
11	1	-0.897946000	2.482368000	0.803074000
12	7	-2.470022000	0.600536000	-0.208885000
13	6	3.897396000	-0.476265000	-0.311423000
14	1	3.634777000	-0.987347000	-1.245386000
15	1	4.923793000	-0.116929000	-0.409590000
16	6	3.765572000	-1.398242000	0.891483000
17	1	4.423598000	-2.264339000	0.768211000
18	1	4.056850000	-0.864727000	1.799933000
19	1	2.741144000	-1.756991000	1.012959000
20	1	-2.873262000	1.502493000	0.008136000
21	6	-3.263528000	-0.493993000	0.121592000
22	8	-2.842155000	-1.619804000	0.314945000
23	7	-4.612058000	-0.150516000	0.233702000
24	1	-4.946200000	0.560584000	-0.402025000
25	1	-5.206481000	-0.967239000	0.265857000

Table S14. Cartesian coordinates for conformer X of dulcin at the MP2/6-311++G(d,p) level.

Standard orientation

Center Number	Atomic Number	X	Y	Z
1	6	-0.326523000	-0.765900000	0.094434000
2	6	1.047721000	-0.754382000	-0.176369000
3	6	-1.037154000	0.436311000	0.189925000
4	1	-0.844268000	-1.710856000	0.206144000
5	6	1.735476000	0.460511000	-0.312892000
6	1	1.563104000	-1.704552000	-0.261271000
7	6	1.025353000	1.664180000	-0.193923000
8	8	3.067953000	0.583471000	-0.603059000
9	6	-0.348276000	1.650979000	0.041918000
10	1	1.564552000	2.600165000	-0.307282000
11	1	-0.889864000	2.592688000	0.111168000
12	7	-2.417679000	0.465481000	0.504000000
13	6	3.894509000	-0.564949000	-0.401021000
14	1	3.499243000	-1.420795000	-0.961215000
15	1	4.848706000	-0.286568000	-0.852979000
16	6	4.077136000	-0.891259000	1.073788000
17	1	4.765419000	-1.735284000	1.184641000
18	1	4.497812000	-0.027090000	1.594338000
19	1	3.128575000	-1.153667000	1.547280000
20	1	-2.776031000	1.380351000	0.744286000
21	6	-3.348119000	-0.400512000	-0.061592000
22	8	-3.067244000	-1.361860000	-0.753938000
23	7	-4.660698000	-0.027509000	0.234706000
24	1	-4.815533000	0.376105000	1.148258000
25	1	-5.319478000	-0.759058000	0.006054000

Table S15. Cartesian coordinates for conformer XI of dulcin at the MP2/6-311++G(d,p) level.

Standard orientation

Center Number	Atomic Number	X	Y	Z
1	6	0.582806000	-0.778267000	-0.709629000
2	6	-0.806936000	-0.913749000	-0.773231000
3	6	1.142800000	0.430142000	-0.268057000
4	1	1.226872000	-1.605185000	-0.983015000
5	6	-1.642668000	0.156354000	-0.436194000
6	1	-1.255594000	-1.843920000	-1.111382000
7	6	-1.085805000	1.371344000	-0.021600000
8	8	-3.011434000	0.015181000	-0.545475000
9	6	0.302458000	1.503227000	0.067177000
10	1	-1.738346000	2.206037000	0.220297000

11	1	0.736032000	2.444229000	0.400985000
12	7	2.544096000	0.623477000	-0.229496000
13	6	-3.626037000	-0.244985000	0.724067000
14	1	-3.196551000	-1.161846000	1.150442000
15	1	-3.412380000	0.584176000	1.411763000
16	6	-5.116184000	-0.391665000	0.497036000
17	1	-5.620632000	-0.591945000	1.447023000
18	1	-5.527220000	0.525339000	0.068160000
19	1	-5.316525000	-1.219340000	-0.187329000
20	1	2.835987000	1.583849000	-0.104017000
21	6	3.430628000	-0.330135000	0.264912000
22	8	3.120517000	-1.464456000	0.578726000
23	7	4.725888000	0.174120000	0.389289000
24	1	5.018022000	0.835786000	-0.316627000
25	1	5.401413000	-0.559765000	0.552257000

Table S16. Cartesian coordinates for conformer XII of dulcin at the MP2/6-311++G(d,p) level.

Standard orientation

Center Number	Atomic Number	X	Y	Z
1	6	-0.470123000	-0.534754000	-0.983629000
2	6	0.914978000	-0.650780000	-1.125242000
3	6	-1.005462000	0.517430000	-0.225792000
4	1	-1.129232000	-1.267633000	-1.433460000
5	6	1.774250000	0.296045000	-0.556619000
6	1	1.347166000	-1.467816000	-1.696062000
7	6	1.239951000	1.369146000	0.168029000
8	8	3.128304000	0.167540000	-0.776283000
9	6	-0.143595000	1.468028000	0.341477000
10	1	1.895008000	2.126384000	0.589103000
11	1	-0.556026000	2.293049000	0.919638000
12	7	-2.403730000	0.687300000	-0.084815000
13	6	3.905983000	0.056813000	0.425530000
14	1	4.942634000	0.056860000	0.081714000
15	1	3.756414000	0.942420000	1.055337000
16	6	3.584437000	-1.212784000	1.197336000
17	1	4.229162000	-1.284741000	2.079231000
18	1	2.543045000	-1.213903000	1.530700000
19	1	3.750797000	-2.091342000	0.568184000
20	1	-2.687867000	1.589657000	0.273444000
21	6	-3.265741000	-0.364239000	0.217315000
22	8	-2.939377000	-1.536339000	0.249546000
23	7	-4.554853000	0.082750000	0.510259000
24	1	-5.221215000	-0.676890000	0.530031000
25	1	-4.879297000	0.884579000	-0.012498000

Table S17. Cartesian coordinates for conformer XIII of dulcin at the MP2/6-311++G(d,p) level.

Standard orientation

Center Number	Atomic Number	X	Y	Z
1	6	-0.471109000	-0.971205000	0.228220000
2	6	0.871072000	-1.234077000	-0.057660000
3	6	-0.976154000	0.326816000	0.059266000
4	1	-1.121979000	-1.765480000	0.572862000
5	6	1.723848000	-0.216031000	-0.498391000
6	1	1.276945000	-2.234423000	0.063941000
7	6	1.221583000	1.080552000	-0.669400000
8	8	3.024364000	-0.549021000	-0.806634000
9	6	-0.122231000	1.345496000	-0.389534000
10	1	1.861765000	1.874889000	-1.041606000
11	1	-0.514615000	2.349351000	-0.542638000
12	7	-2.313194000	0.652813000	0.390873000
13	6	4.015551000	0.238044000	-0.131497000
14	1	4.964817000	-0.097798000	-0.554408000
15	1	3.892353000	1.299539000	-0.378140000
16	6	3.982432000	0.026585000	1.373820000
17	1	4.782662000	0.603747000	1.848309000
18	1	3.026623000	0.353587000	1.792388000
19	1	4.124417000	-1.031312000	1.610563000
20	1	-2.511098000	1.644506000	0.417592000
21	6	-3.399311000	-0.152387000	0.055699000
22	8	-3.311169000	-1.272376000	-0.412066000
23	7	-4.617342000	0.486309000	0.293689000
24	1	-4.652956000	1.095695000	1.099182000
25	1	-5.394829000	-0.157741000	0.246469000
